# The Beneficial Effects of Eccentric Exercise in the Management of Lateral Elbow Tendinopathy: A Systematic Review and Meta-Analysis

**DOI:** 10.3390/jcm10173968

**Published:** 2021-09-01

**Authors:** Seo Yeon Yoon, Yong Wook Kim, In Soo Shin, Seok Kang, Hyun Im Moon, Sang Chul Lee

**Affiliations:** 1 Department of Physical Medicine and Rehabilitation, Korea University Guro Hospital, Seoul 08308, Korea; seoyeon0521@gmail.com (S.Y.Y.); caprock@paran.com (S.K.); 2 Department and Research Institute of Rehabilitation Medicine, Yonsei University College of Medicine, Seoul 03722, Korea; ywkim1@yuhs.ac; 3 Department of Transdisciplinary Security, Dongguk University, Seoul 04620, Korea; 9065031@hanmail.net; 4 Department of Rehabilitation Medicine, Bundang Jesaeng General Hospital, Gyeonggi-do 13590, Korea; feellove99@gmail.com

**Keywords:** eccentric exercise, lateral elbow tendinopathy, tennis elbow, pain, function

## Abstract

As a first-line treatment for lateral elbow tendinopathy (LET), eccentric exercise has been suggested as a conservative treatment method. This study aimed to investigate the impact of eccentric exercise on LET with regard to pain reduction, and strength and functional improvement. The PubMed, EMBASE, and Cochrane Central Register of Controlled Trials databases were searched, and studies up to May 2021 were included if (1) randomization was used for patient allocation, (2) the study comprised patients with LET, (3) the intervention was eccentric exercise, and (4) the primary outcomes included improvement in pain intensity, muscle strength, or function. The meta-analysis comprised of six studies, totaling 429 participants. Additional eccentric exercise with underlying adjuvant therapy significantly improved the visual analog scale (VAS) scores (standardized mean difference [SMD], −0.63; 95% confidence interval [CI], −0.90–−0.36) and muscle strength (SMD, 1.05; 95% CI, 0.78–1.33) compared with adjuvant therapy alone. Compared with the concentric or isotonic exercise group, the eccentric exercise group showed significantly improved VAS scores (SMD, −0.30; 95% CI, −0.58–−0.02). However, no differences in muscle strength and function were observed between the two groups. Eccentric exercise can improve pain and muscle strength in patients with LET. The limited number of included studies and heterogeneous exercise parameters are important when interpreting these findings.

## 1. Introduction

Lateral elbow tendinopathy (LET) is one of the most frequently observed lesions causing work- or sports-related pain disorders [1]. LET is associated with pain in the lateral epicondyle, attributed to impaired tendon healing, rather than inflammatory or degenerative changes [2,3]. LET most commonly affects the structure of the extensor carpi radialis brevis [4]. The prevalence of LET peaks between the ages of 30 and 60 years and LET primarily affects the dominant arm [2], with symptoms appearing for a longer duration and with greater severity in women [3,5]. Given the variety of symptoms experienced (i.e., pain and loss of function), patients often withdraw from many daily activities, including work and sports. The costs due to loss of work and reduced daily activities, are often considerable, owing to the long recovery period [6].

For the treatment of LET, many non-surgical options exist that focus on rest, physical therapy, and interventions such as the use of a brace, drugs (including corticosteroid injections), and acupuncture. However, open surgical debridement of the involved area is often required [7]. Debate continues over the best strategy for the management of LET.

It has been suggested that eccentric exercise programs should be used as a first-line conservative treatment for LET in order to lengthen the musculotendinous unit while applying a load [8,9,10,11]. While effective in treating tendinopathy, the mechanisms through which eccentric exercise functions remain unclear [12,13]. Although not fully investigated, several hypothetical mechanisms have been proposed to explain the methods by which eccentric loading influences the tendon, inducing structural tendon adaptation [14,15], tendon length changes [15], reduction in neuro–vascular ingrowth [16,17,18], alteration in tenocyte activity [19,20], and force fluctuation within the tendon [21,22,23,24]. Eccentric exercise appears to induce significant positive changes in LET lesions, but the clinical importance of these changes has not been proven.

To our knowledge, only two systematic reviews [25,26] assessed the effectiveness of eccentric exercise in LET, and no meta-analysis has been performed on the efficacy of eccentric exercise in LET. In many previous studies on the treatment of LET, multimodal therapy was performed, which made it difficult to isolate the effects of eccentric exercise on LET [27,28,29]. Therefore, the objective of this study was to clarify the current evidence on the effect of eccentric exercise on pain reduction, and strength and functional improvement in patients with LET by performing a novel systematic review and meta-analysis of all currently available data. We compared the effect of eccentric exercise and adjuvant therapy with that of the same adjuvant therapy alone in order to clarify the effect of eccentric exercise. In addition, we compared the effects of eccentric exercise with those of other strengthening exercises such as concentric or isotonic exercises.

## 2. Materials and Methods

This systematic review was performed in accordance with the Preferred Reporting Items for Systematic Reviews and Meta-Analyses guidelines. Study selection, eligibility criteria application, data extraction, and statistical analysis adhered to the Cochrane Collaboration guidelines [30,31].

### 2.1. Search Strategy and Study Selection

From the public databases MEDLINE (PubMed), EMBASE, and Cochrane Central Register of Controlled Trials, we retrieved relevant studies up to May 2021 with no date restrictions. Searches were restricted to articles published in English or Korean. The key terms used to retrieve articles from the selected databases were combined with the following English terms and their equivalents: “eccentric exercise” AND “lateral elbow tendinopathy”. The search methods used for each database are outlined in Supplementary File S1 (available online). Studies were included if: (1) patient allocation was randomized; (2) the sample was composed of patients with LET; (3) the intervention was eccentric exercise; and (4) the study outcome was pain intensity, strength, or function. Studies were excluded if: (1) the trial did not have an appropriate comparison group; (2) eccentric exercise was performed with other non-surgical treatments and the effects of eccentric exercise could not be isolated; or (3) data on pain intensity, strength, or function were not sufficiently reported. We only included studies in which the effects of eccentric exercise could be evaluated (eccentric exercise + adjuvant therapy vs. same adjuvant therapy) or (eccentric exercise vs. other strengthening exercises). The following media types were excluded: abstracts and conference proceedings lacking sufficient reporting details, editorials, review articles, and other nonclinical trials.

The selected studies were independently reviewed for eligibility by two reviewers (SYY and YWK) who assessed the titles and abstracts of the articles. Articles that clearly failed to meet the inclusion criteria were not reviewed further; however, for those that could not be easily excluded, the full texts were retrieved and reviewed before determining inclusion. Regarding inclusion, differences in opinion between reviewers were resolved through discussion with a third author (SCL). Full texts of studies that met the inclusion criteria were retrieved and reviewed in detail.

### 2.2. Data Extraction and Quality Assessment

From the included studies, we extracted the following information: first author, year of publication, patient demographics and clinical presentations, exercise protocols, total sample size and sample size per group (eccentric exercise and comparison groups), methodology used to evaluate the efficacy of the interventions, baseline and end-point outcome measurements, and adverse events. The variables of interest were methods used to gauge pain intensity, such as the visual analog scale (VAS) score; muscle strength (including grip strength); and function using the Disability of Arm, Shoulder, and Hand questionnaire (DASH). For continuous outcomes, if the median and range were reported, the mean and standard deviation were calculated based on a previous study [32]. In studies where only graphs were published, data values were determined using a plot digitizer.

To assess study quality, we used the Cochrane risk-of-bias tool to rate the risk of selection bias, performance bias, detection bias, attrition bias, reporting bias, and other sources of bias [30]. The reviewers (SYY and YWK) independently assessed the risk of bias for each of the following domains (i.e., low, unclear, or high risk of bias): “sequence generation”, “allocation concealment”, “blinding”, “incomplete outcome data addressed”, “selective outcome reporting”, or “other bias”. Any discrepancies in the initial ratings of the methodological quality assessment were resolved by discussion with a third author (SCL).

### 2.3. Data Synthesis and Statistical Analysis

To represent the quantitative findings of each study, a meta-analysis uses effect sizes, which produce a statistical standardization for comparison between studies [33]. To calculate the effect sizes of eccentric exercise on LET, changes were based on the standardized mean difference (SMD). The mean VAS pain score, mean strength including grip strength, mean function, standard deviations prior to and following treatments, and the SMD were calculated using the total number of patients in the treatment and control groups. The SMD and 95% confidence interval (CI) for each study were calculated by comparing the treatment and comparison groups, after which the results were pooled. Because the number of studies included was small, we used a fixed effect model according to Borenstein et al. [34]. The RevMan 5.3 software (The Nordic Cochrane Center, The Cochrane Collaboration, Copenhagen, Denmark) was used to conduct the analyses. The degree of heterogeneity was assessed using *I*^2^ statistics. Expressed as a percentage ranging from 0 to 100, these statistics indicate the degree of heterogeneity in the system, as a measure of total variation between study variances [35]. To determine the stability and reliability of the pooled findings, a sensitivity analysis was performed by excluding each study individually and reanalyzing the remaining studies. We used two-tailed *p* values, and statistical significance was set at *p* < 0.05.

## 3. Results

### 3.1. Description of the Selected Studies

**Figure 1:**
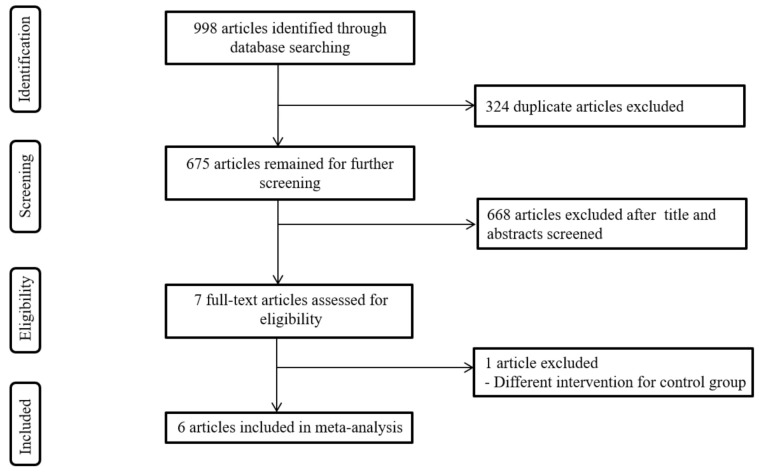
Flow chart of study search and selection methods.

A diagrammatic representation of the search process is shown in Figure 1. A total of 998 studies were initially identified using the search criteria. After removing duplicates, 675 studies remained. Based on the results of the screening of titles and abstracts, 668 studies were discarded. The full texts of the seven remaining papers were retrieved and assessed in more detail for eligibility, and one study was excluded as the intervention protocol for the comparison group was inconsistent with the inclusion criteria. Finally, six studies [36,37,38,39,40,41] with a total of 429 participants, were included in the meta-analysis.

### 3.2. Risk of Bias

**Figure 2:**
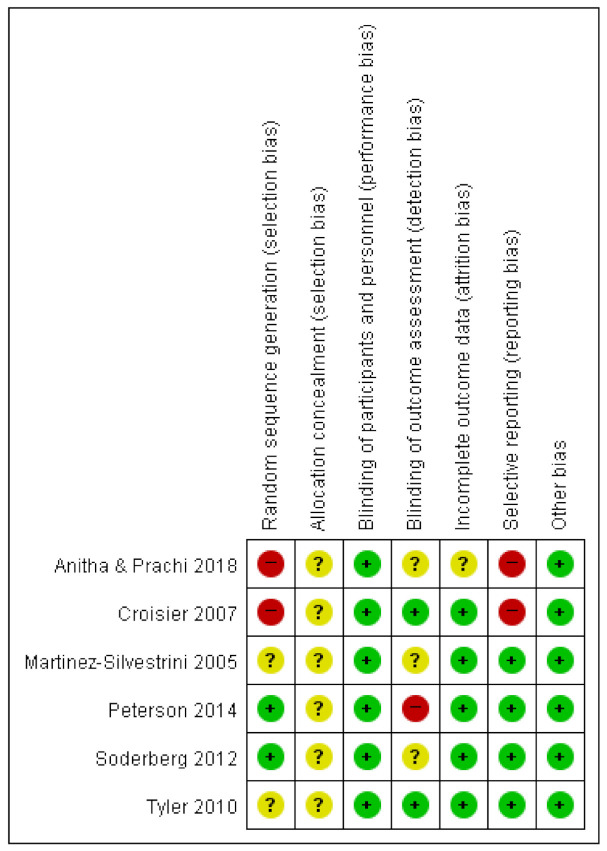
Risk of bias summary.

Two studies [36,37] were determined to have a high risk of random sequence generation because they used methods of convenient sampling or allocation according to the judgment of the clinician. Two studies [38,41] provided insufficient details regarding how randomization was performed and were determined to have an unclear risk of bias. In all six studies [36,37,38,39,40,41], the methods did not describe the allocation concealment, and all studies were determined to have an unclear risk. As these studies were experimental trials of performing eccentric exercises by the participants themselves, complete participant blinding seemed difficult. In this study, we only included studies with non-surgical interventions, such as strengthening, stretching, or physical modalities, in both the experimental and comparison groups. Thus, we regarded these studies as having a low risk of participant blinding. One study [39] with an unblinded assessor was considered to have a high risk of assessment blinding. A high risk of selective reporting was ascribed to two studies [36,37] that did not report the scales of pre-intervention. We only included studies from which the effects of eccentric exercise could be evaluated: (eccentric exercise + adjuvant therapy vs. same adjuvant therapy) or (eccentric exercise vs. other strengthening exercises). Thus, there was a low risk of other biases regarding interventions. Figure 2 provides a detailed evaluation of the methodological quality.

### 3.3. Study Characteristics

The number of participants in the included studies ranged from 21 [41] to 120 [39]. The average age of the participants ranged from 38 [37] to 51 [41] years. Four of the studies [37,39,40,41] clearly identified participants of both sexes; however, the remaining studies failed to report participant sex [36,38]. Four studies [36,37,38,40] compared the effects of eccentric exercise and adjuvant therapy versus the same adjuvant therapy, and three studies [38,39,41] evaluated the effects of eccentric exercise in comparison to other exercises such as concentric or isotonic exercise. The devices used for eccentric exercises differed across studies. They included rubber bars [36,41], elastic bands [38], plastic water containers [39], buckets [40], and Cybex Norm [37]. Exercise protocols with two or three sets of 10 or 15 repetitions were commonly used. The frequency of exercise ranged from three times a week [37] to twice a day [40], and the duration of intervention ranged from 2 weeks [36] to 3 months [39]. In only one study [39], follow-up evaluation was performed at 3 and 9 months, whereas in the other studies, follow-up evaluation was not performed after completion of the intervention. All six [36,37,38,39,40,41] included studies reported pain intensity as an outcome variable. Grip strength was reported in three studies [36,38,40], and extensor muscle strength was reported in two studies [37,39]. DASH outcome measures were presented in three studies [38,39,41]. Table 1 outlines the characteristics of the included studies.

### 3.4. Add-on Effects of Eccentric Exercise with Adjuvant Therapy for LET

**Figure 3:**
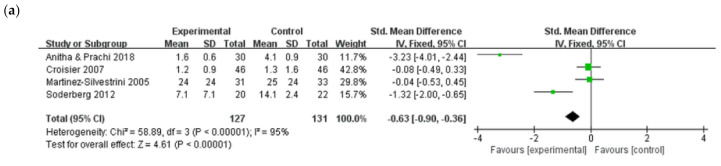

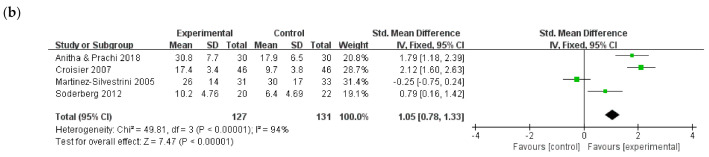
Add-on effects of eccentric exercise with adjuvant therapy on (**a**) pain intensity and (**b**) muscles strength in lateral elbow tendinopathy.

The four studies that compared eccentric exercise and adjuvant therapy with the same adjuvant therapy alone reported that the VAS score reflects pain intensity [36,37,38,40]. A significant improvement in the VAS score after eccentric exercise (SMD, −0.63; 95% CI, −0.90, −0.36) relative to the VAS score in the comparison group was observed in the meta-analysis. Four studies [36,37,38,40] reported outcomes of muscle strength: three studies [36,38,40] with grip strength and one study [37] with eccentric muscle strength. A significant improvement in muscle strength in the eccentric exercise group (SMD, 1.05; 95% CI, 0.78–1.33) relative to the comparison group was observed in the meta-analysis. Sensitivity analysis, conducted by individually excluding the studies, also showed beneficial effects of eccentric exercise in pain reduction and muscle strength improvement in patients with LET (Figure 3).

### 3.5. Comparison between Eccentric Exercise and Other Exercises (Concentric or Isotonic) in LET

**Figure 4:**
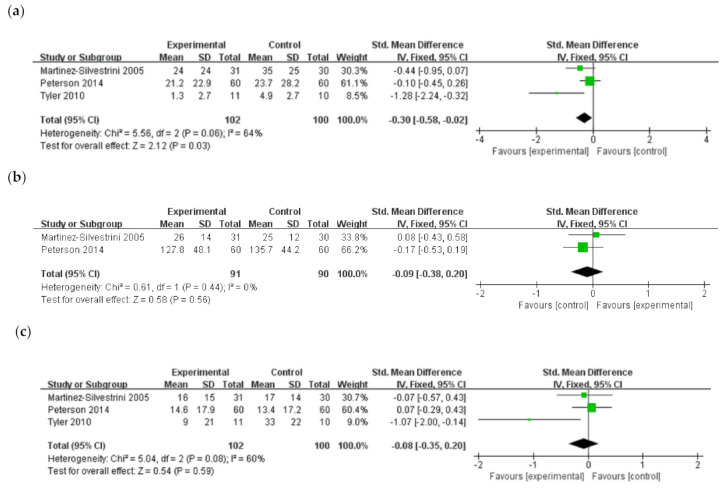
Effects of eccentric exercise compared with those of other exercise (concentric or isotonic) on (**a**) pain intensity, (**b**) muscles strength, and (**c**) function in lateral elbow tendinopathy.

Three studies [38,39,41] compared the effects of eccentric exercises with those of other strengthening exercises such as concentric or isotonic exercise. There was a significant improvement in pain intensity after eccentric exercise (SMD, −0.30; 95% CI, −0.58–−0.02) relative to other exercises. Of the three studies [38,39,41], two [38,39] evaluated muscle strength and there was no significant difference in muscle strength between the two groups (SMD, −0.09; 95% CI, −0.38–0.20). Function was evaluated using the DASH results in three studies [38,39,41], and the meta-analysis did not reveal any significant difference in functional improvement (SMD, −0.08; 95% CI, −0.35, 0.20) between the two groups (Figure 4).

### 3.6. Adverse Events

Of the six studies [36,37,38,39,40,41] included in this review, only one study [40] reported no adverse events. Another study [37] reported that there was no delayed-onset muscle soreness in the eccentric exercise group. There were no reports of adverse events during the study period in four studies [36,38,39,41].

## 4. Discussion

To our knowledge, this is the first meta-analysis to evaluate the effectiveness of eccentric exercise on pain reduction, and muscle strength and functional improvement in patients with LET. We reviewed six studies that included a total of 429 participants with LET, and the meta-analysis revealed that eccentric exercise might improve outcomes regarding pain and muscle strength in these patients. Eccentric exercise combined with adjuvant therapy showed beneficial effects on pain reduction and muscle strength improvement. Comparison between eccentric exercise and other exercises showed the positive effects of eccentric exercise on pain reduction; however, there was no significant difference in strength and function between the groups. Due to the small number of included studies and differing parameters of eccentric exercise, we cannot report the estimated effects with confidence, and suggest beneficial effects of eccentric exercise on LET with limited evidence.

The pathogenesis of tendinopathy could be due to repetitive tensile loads that exceed the mechanical strength of the tendon [15]. Therefore, eccentric exercise has been performed in various tendinopathies, including Achilles and patellar tendinopathies. Eccentric contraction creates a greater stimulus in the cells of the tendon, resulting in increased cross-linking of collagen [19,20]. Decreased neovascularization has been suggested as another benefit of eccentric exercise, resulting in pain reduction [17,18]. In many previous studies, eccentric exercise was performed in combination with other conventional adjuvant treatments such as ultrasound, brace, ice, or massage, which made it difficult to isolate the effects of eccentric exercise. Thus, many systematic reviews of eccentric exercise for chronic tendinopathy could not pool the results and reported only limited evidence on the beneficial effects on pain reduction or function improvement. A recent meta-analysis evaluating the impact of heavy eccentric calf training of the mid-portion Achilles tendon included seven studies for quantitative analysis and suggested positive effects with limited confidence.

Eccentric exercise programs have also been applied to LET based on the similar mechanism of tendinopathies such as Achilles and patellar tendinopathies. There have been two systematic reviews on the effects of eccentric exercise in LET with promising results [25,26]. One systematic review in 2008 reported encouraging results with the inclusion of only four studies [26], and another review in 2014 suggested that including eccentric exercise in a multimodal therapy program improves outcomes in patients with LET [25]. In these two reviews, the included studies were too heterogeneous to pool the results. The protocols for eccentric exercise varied across the studies, the comparison group received different treatments (Cyriax physiotherapy, ultrasound, or iontophoresis) across the studies, or eccentric exercise was applied alone or combined with other adjuvant therapy to the experimental group. These methodological differences made it difficult to isolate the effects of eccentric exercise and pool the results for evidence of benefits of eccentric exercise in LET. Therefore, in this study, we only included studies from which the effects of eccentric exercise could be isolated (eccentric exercise + adjuvant therapy vs. same adjuvant therapy) or (eccentric exercise vs. other strengthening exercises) and included six studies in the final meta-analysis.

The addition of eccentric exercise to adjuvant therapy in LET resulted in beneficial effects with regard to pain reduction and increase in muscle strength. However, we could not conclude that these positive effects originated solely from eccentric exercise or were combined effects with other adjuvant therapies. Conventional adjuvant therapies such as ultrasound, massage, or stretching are easily applied and have relatively low side effects; therefore, we believe that evaluation of the effects of additional eccentric exercise with conventional adjuvant therapy rather than direct comparison of eccentric exercise with conventional therapy would provide more clinically relevant information. On comparing eccentric and other strengthening exercises, eccentric exercise showed better effects on pain reduction compared to other strengthening exercises, which is in line with previous studies on Achilles tendinopathy [42]. The possible mechanism suggested was that eccentric loading might be associated with certain metabolic changes within the tendon, which cause an alteration in the pain perception by the tendon [42].

The optimal parameter for eccentric exercise has not yet been determined. Thus, the interventional protocols used in the included studies varied across studies. Five studies used devices such as rubber bars [36,41], elastic bands [38], plastic water containers [39], and buckets [40], which can be used in home-based exercise programs, whereas one study used Cybex Norm [37] in a hospital-based exercise program. Eccentric exercise in hospital-based programs showed beneficial effects in pain reduction and functional improvement in LET [37]; however, the outcomes of home-based exercise programs differed across the studies irrespective of supervision. Moreover, two studies with home-based eccentric exercise without supervision showed the positive effects of eccentric exercise [39,41]. Few and simple exercises that could increase adherence to treatment are important for successful outcomes in home-based exercise training [43], and the effects of supervision during exercise need to be further investigated. Another issue is the relationship between exercise intensity and pain. Whether eccentric exercises should be performed with pain remains unclear. Pain during eccentric exercise is thought to be due to a direct mechanical influence on the tendon’s neural supply which may facilitate pain reduction with exercise that induces moderate pain leading to favorable outcomes in Achilles tendinopathy [14]. In three of the studies included in the meta-analysis, patients performed eccentric exercise with pain-free intensity [37,38,40], while the other three studies did not exactly report the relationship of exercise intensity to pain and, based on methodology description, patients seemed to perform exercise with only discomfort at best [36,39,41]. Pain during exercise could decrease compliance with exercise (especially in unsupervised settings), resulting in a high dropout rate. There was no delayed-onset muscle soreness [40], and there seemed to be no other serious adverse events related to exercise in the included studies, although there was limited description of adverse events. Future studies on the magnitude of pain during exercise and the feasibility of better outcomes in LET are recommended.

This study had several limitations. First, only a limited number of studies and participants were included in this review. Many studies on the treatment of LET evaluated multimodal therapy, which made it difficult to isolate the effects of eccentric exercise on LET. We wanted to determine the effects of eccentric exercise on LET; thus, we included only studies in which the effects of eccentric exercise could be evaluated. Second, the diversity of exercise parameters utilized by individual studies may have caused variations in the results. The device, intensity, protocol, and presence of supervision differed across studies. With these various parameters and the low number of included studies, we could not perform subgroup analysis and draw conclusions on the optimal protocols of eccentric exercise on LET. Third, exercise intensity and related adverse events were not explicitly described in some of the included studies; thus, we could not elucidate effective and safe exercise intensity. Although our analysis suggested favorable effects of eccentric exercise, the low number of studies with various exercise parameters made it challenging to be confident of the effects of eccentric exercise on LET. Future studies with an optimal protocol and device for eccentric exercise, and study design in which the effects of eccentric exercise could be isolated, are recommended.

## 5. Conclusions

In this meta-analysis, eccentric exercise combined with adjuvant therapy showed beneficial effects with regard to pain reduction and muscle strength improvement. Comparison between eccentric exercise and other exercises showed positive effects of eccentric exercise with regard to pain reduction; however, the differences in muscle strength and function between the groups were not significant. Due to the small number of included studies and various parameters of eccentric exercise, we could not be confident in the estimated effects, and suggested beneficial effects of eccentric exercise on LET with limited evidence. Future studies with an optimal protocol and device for eccentric exercise, with a study design in which the effects of eccentric exercise can be isolated, are recommended.

## Figures and Tables

**Table 1:** Characteristics of the included studies.

	Participants (E/C)	Eccentric Exercise Group	Comparison Group	Outcome Measures
Eccentric exercise + adjuvant treatment vs. adjuvant treatment				
Anitha and Prachi (2018) [36]	*N* = 30/30 Age (y), NR/NR Sex (M/F), NR/NR Duration (mo), NR/NR	Eccentric exercise + Standardized conventional therapy Device: Rubber bar 3 sets of 15 repetitions 6 session/week Duration, 2 weeks	Standardized conventional therapy (pulsed ultrasonic therapy)	Pain: Numerical pain rating scale (0–10) Grip strength (maximum)
Croisier et al. (2007) [37]	*N* = 46/46 Age (y), 40 (SD, 8)/38 (SD, 8) Sex (M/F), 36/56 Duration (mo), 8 (SD, 4)/8 (SD, 3)	Isokinetic eccentric exercise + Non-strengthening rehabilitation Device: Cybex Norrn dynamometer 2 sets of 10 repetitions for wrist extensors and forearm supinators 3 times/week Duration, 9 weeks	Non-strengthening rehabilitation (e.g., ultrasound, analgesic TENS, ice, deep friction massage, and stretching)	Pain: VAS score (0–10) Eccentric muscle strength Disability questionnaire
Martinez-Silvestrini et al. (2005) [38]	*N* = 31/33 Age (y), 46.6 (SD, NR)/43.1 (SD, NR) Sex (M/F), NR/NR Duration (mo), NR/NR	Eccentric exercise + Self-applied ice massage and stretching of wrist extensors Device: Elastic resistance band 3 sets of 10 repetitions Daily Duration, 6 weeks	Self-applied ice massage and stretching of wrist extensors	Pain: VAS score (0–100) Grip strength (pain-free) DASH PRFEQ SF-36
Soderberg et al. (2012) [40]	*N* = 20/22 Age (y), 48 (SD, 12.4)/50 (SD, 10.8) Sex (M/F), 24/18 Duration (mo), NR/NR	Eccentric exercise + Forearm band and warm-up exercise Device: Bucket 2→3 sets of 8–12 repetitions Daily → Twice a day Duration, 6 weeks	Forearm band and warm-up exercise for wrist extensors (flexion, extension, abduction, adduction and circumduction for 1 min twice a day)	Pain: VAS score (0–100) Grip strength (pain-free)
Eccentric exercise vs. Other exercises (concentric or isotonic)				
Martinez-Silvestrini et al. (2005) [38]	*N* = 31/30 Age (y), 46.6 (SD, NR)/47.0 (SD, NR) Sex (M/F), NR/NR Duration (mo), NR/NR	Eccentric exercise + Self-applied ice massage and stretching of wrist extensors Device: Elastic resistance band 3 sets of 10 repetitions Daily Duration, 6 weeks	Concentric exercise + Self-applied ice massage and stretching of wrist extensors Same protocol with eccentric exercise	Pain: VAS score (0–100) Grip strength (pain-free) DASH PRFEQ SF-36 results
Peterson et al. (2014) [39]	*N* = 60/60 Age (y), 48.8 (SD, 6.7)/47.0 (SD, 9.4) Sex (M/F), 63/57 Duration (mo), NR/NR	Eccentric exercise Device: Dumbbell (using a specified amount of water in a plastic container) 3 sets of 15 repetitions DailyDuration, 3 months	Concentric exercise Same protocol with eccentric exercise	Pain: VAS score (0–100) Forearm extensor muscles strength DASH Gothenburg Quality of Life instrument questionnaire
Tyler et al. (2010) [41]	*N* = 11/10 Age (y), 47 (SD, 2)/51 (SD, 4) Sex (M/F), 10/11 Duration (wk), 6 (SD, 2)/8 (SD, 3)	Eccentric exercise + Wrist extensor stretching, ultrasound, cross-friction massage, heat, and ice Device: Rubber bar 3 sets of 15 repetitions Daily Duration, 7 weeks	Isotonic exercise + Wrist extensor stretching, ultrasound, cross-friction massage, heat, and ice	Pain: VAS score (0–10) DASH

E, experimental; C, comparison; M, male; F, female; SD, standard deviation; NR, not reported; VAS, visual analog scale; DASH, disabilities of the arm, shoulder, and hand; PRFEQ, patient-rated forearm evaluation questionnaire SF-36, short form-36.

## Data Availability

The datasets used and/or analyzed in the current study are available from the corresponding author upon reasonable request.

## Supplementary Materials

The following are available online at https://www.mdpi.com/article/10.3390/jcm10173968/s1, Supplementary File S1: Search strategies.
