# Supplementary material for: The Beneficial Effects of Eccentric Exercise in the Management of Lateral Elbow Tendinopathy: A Systematic Review and Meta-Analysis
Source: J Clin Med. 2021 Sep 1;10(17):3968. doi: 10.3390/jcm10173968 (PMC8432114; doi:10.3390/jcm10173968)
Supplement: Supplementary file 1 [file jcm-10-03968-s001.zip › jcm-1331996-SI.pdf]

Supplementary material

Appendix 1. Search strategies

PubMed

Recent queries in pubmed		
Search	Query	Items found
#8	#4 AND #7	331
#7	#5 OR #6	438307
#6	Search exercise[MeSH Terms]	210808
#5	Search "eccentric exercise" OR eccentric OR exercise OR strengthening	325467
#4	#1 OR #2 OR #3	5782
#3	Search tennis elbow[MeSH Terms]	1728
#2	Search elbow tendinopathy[MeSH Terms]	1758
#1	Search ("lateral epicondylitis" OR "elbow pain" OR “tennis elbow” OR epicondyl* OR “elbow tend*”)	5536

EMBASE

No.	Query	Results
#3	#1 AND #2	417
#2	strengthening exercise'/exp OR 'eccentric exercise'/exp OR 'eccentric exercise':ab,ti OR eccentric:ab,ti OR exercise:ab,ti OR strengthening:ab,ti	423877
#1	lateral epicondylitis'/exp OR 'elbow pain'/exp OR 'tennis elbow'/exp OR 'lateral epicondylitis':ab,ti OR 'elbow pain':ab,ti OR 'tennis elbow':ab,ti OR epicondyl*:ab,ti OR 'elbow tend*':ab,ti	7788

Cochrane Central Register of Controlled Trials (CENTRAL)

#1 "lateral epicondylitis" OR "elbow pain" OR “tennis elbow” OR epicondyl* OR “elbow tend*” 1320

#2 MeSH descriptor: [Tennis Elbow] explode all trees 400

#3 MeSH descriptor: [Elbow Tendinopathy] explode all trees 404

#4 #1 OR #2 OR #3 1321

#5 "eccentric exercise" OR eccentric OR exercise OR strengthening 109388

#6	MeSH descriptor: [Exercise] explode all trees	25628
#7	#5 OR #6	112453
#8	#4 AND #7	276
